# Bioinspired preactivation reflex increases robustness of walking on rough terrain

**DOI:** 10.1038/s41598-023-39364-3

**Published:** 2023-08-14

**Authors:** Elsa K. Bunz, Daniel F. B. Haeufle, C. David Remy, Syn Schmitt

**Affiliations:** 1 Institute for Modelling and Simulation of Biomechanical Systems, University of Stuttgart, Stuttgart, Germany; 2 Stuttgart Center for Simulation Science, University of Stuttgart, Stuttgart, Germany; 3 Institute of Computer Engineering, Heidelberg University, Heidelberg, Germany; 4 Hertie Institute for Clinical Brain Research and Center for Integrative Neuroscience, Tuebingen, Germany; 5 Center for Bionic Intelligence Tuebingen Stuttgart, Tuebingen Stuttgart, Germany; 6 Institute for Nonlinear Mechanics, University of Stuttgart, Stuttgart, Germany

**Keywords:** Biomedical engineering, Computational biophysics, Spinal cord, Reflexes, Biophysical models

## Abstract

Walking on unknown and rough terrain is challenging for (bipedal) robots, while humans naturally cope with perturbations. Therefore, human strategies serve as an excellent inspiration to improve the robustness of robotic systems. Neuromusculoskeletal (NMS) models provide the necessary interface for the validation and transfer of human control strategies. Reflexes play a crucial part during normal locomotion and especially in the face of perturbations, and provide a simple, transferable, and bio-inspired control scheme. Current reflex-based NMS models are not robust to unexpected perturbations. Therefore, in this work, we propose a bio-inspired improvement of a widely used NMS walking model. In humans, different muscles show an increase in activation in anticipation of the landing at the end of the swing phase. This preactivation is not integrated in the used reflex-based walking model. We integrate this activation by adding an additional feedback loop and show that the landing is adapted and the robustness to unexpected step-down perturbations is markedly improved (from 3 to 10 cm). Scrutinizing the effect, we find that the stabilizing effect is caused by changed knee kinematics. Preactivation, therefore, acts as an accommodation strategy to cope with unexpected step-down perturbations, not requiring any detection of the perturbation. Our results indicate that such preactivation can potentially enable a bipedal system to react adequately to upcoming unexpected perturbations and is hence an effective adaptation of reflexes to cope with rough terrain. Preactivation can be ported to robots by leveraging the reflex-control scheme and improves the robustness to step-down perturbation without the need to detect the perturbation. Alternatively, the stabilizing mechanism can also be added in an anticipatory fashion by applying an additional knee torque to the contralateral knee.

## Introduction

Walking over unknown and uneven terrain is an important ability for bipedal robots and vital for the control of exoskeletons and orthoses. Yet, stable and robust locomotion over uneven terrain remains a major challenge for robots^1^. At the DARPA Robotics Challenge, for example, a common failure mode of the bipedal robots was falling on rough terrain^2^. In contrast to robots, humans naturally adapt to different terrain and can efficiently handle a wide range of unexpected perturbations. In their ability, they provide an ample source of inspiration for the development of robust locomotion controllers.

Humans deploy different strategies for perturbation handling^3,4^. *Anticipatory* strategies adapt the step cycle one or two steps before the encounter of the perturbation. They allow for a proactive response tailored to the perturbation but require the detection of the perturbation ahead of time, e.g., through vision. In humans, an example of a common anticipatory strategy is the adaptation of muscles of the contralateral leg, depending on the height of the perceived perturbation^5^. *Predictive* strategies, on the other hand, can be employed without visual perturbation detection. They are triggered by detecting deviations from an expected nominal behavior. For example, if ground contact does not happen at the expected time, a step-down perturbation may be present and a reaction is triggered^6^. Predictive strategies do not require visual perturbation detection yet must have a robust model of the expected nominal sensor inputs. *Accommodation* strategies avoid this complex modelling by adapting the gait at large when moving through rough environments and sustaining these modifications over several steps. In humans, landing more flat-footed^7^ or widening motor primitives^8^ are examples of such strategies. As accommodation strategies are independent of perturbation detection and prediction, they are well suited for deriving control strategies specifically tailored to rough and uneven terrain.

In landing activities, humans exhibit a preparatory muscular activity prior to landing impact^9^ based on impact prediction. Similarly, in our work, preactivation is an activation of leg muscles at the end of the swing phase, prior to touchdown. The moment of touchdown of the swing foot constitutes a critical instant for walking stability due to the occurring impact forces and the irregular nature of ground conditions. With preactivation, the leg is better prepared for landing and can better react to unexpected perturbations^10–13^, especially in the presence of neuronal delays^14^. Preactivation can be observed in humans during running and walking, and proprioceptive information likely contributes to its generation^15–17^. This makes preactivation a promising candidate for developing a bio-inspired accommodation strategy to cope with step-down perturbations.

Neuromusculoskeletal (NMS) models can provide inspiration for robot controller design and provide the necessary interface to explore the applicability of human strategies to robotic systems. While originally developed to study muscle-driven systems, they are equally well suited for the control of torque-actuated robots^18–24^. For example, an NMS model can be used to calculate desired torques given joint state information^23,25^. Such an approach is especially suited for controlling exoskeletons and orthoses, where an understanding of human movement control is fundamental for a natural integration. A model to study perturbation rejection strategies of humans should incorporate proprioception and reflexes, as human gait control relies heavily on proprioceptive information^26,27^. Sensory information and reflexes are also important for detecting and reacting to unexpected perturbations^28–30^.

**Figure 1:**
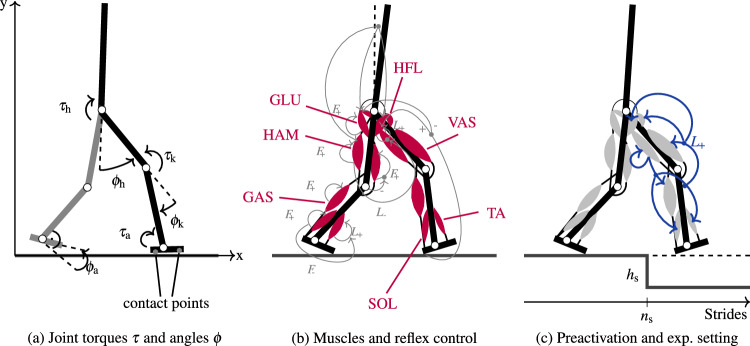
Model, control strategy, and experimental setting. (**a**) The model of Geyer and Herr^31^ contains three joints per leg. Ground contact is modeled with two contact points per foot (heel, toe). (**b**) Each leg of the model is actuated by seven muscles (red), which are stimulated by a set of reflexes (gray) based on trunk angle, ground contact, leg loading, joint angle, force (*F*) and length (*L*) information. (**c**) We add preactivation to the model as an additional reflex, using length information from the hamstrings (blue). The rest of the control remains identical to the model of Geyer and Herr^31^. During the step-down perturbation, the ground is lowered by $h_\text {s}$ after $n_\text {s}$ strides synchronized to the gait cycle. Figures adapted from^31^.

Geyer and Herr^31^ proposed an NMS model that generates walking through a set of reflexes. It models the human body as a trunk and a pair of three-segmented legs (thigh, shank, foot), which are actuated by seven Hill-type muscles per leg. The model produces biological walking dynamics with similar muscle activation patterns as observed in humans when walking on level ground. Moreover, it has been successfully used to control real robotic systems^22,25,32,33^ and in a controller to allow for stair ascent and descent^23,34^. The model is thus well suited to study strategies to improve walking robustness. Notably, the model of Geyer and Herr maximally tolerates unexpected down steps of 3 cm, while humans can cope with markedly higher unexpected step-down perturbations (5 cm^6^ or 10 cm^35^). To improve the robustness of the model, Schreff et al.^36^ proposed an anticipatory strategy. Based on the detection of a perturbation, the contralateral leg before touchdown is determined, and its motion is adapted before the perturbation happens. This strategy allows descending steps of up to 21 cm. Haeufle et al.^37^ employed a predictive strategy to improve the robustness to unexpected step-down perturbations. They added a feed-forward component to the original control scheme that causes additional muscle activation if the ground contact happens later than expected. To predict the expected timing of ground contact, a complex feed-forward signal must be computed and synchronized with the gait. With this addition, the model can cope with step-down perturbations of 7 cm. Both strategies allowed to increase the robustness of the model, based either on (visual) perturbation anticipation or an adequate forward control signal generated by some internal model.

In this paper, we suggest a third option: we propose to use preactivation as an accommodation strategy that prepares the system for unexpected perturbations while moving on rough and unstructured terrain. This strategy has the advantage that it does not require a priori knowledge of a specific perturbation or an internal, synchronized model of the nominal motion. Instead, it can be implemented based on simple proprioceptive sensor signals. The original model of Geyer and Herr^31^ does not include a preparation for stance at the end of the swing phase, and, to our knowledge, the potential effect of preactivation as an accommodation strategy to perturbations has not been studied before. In our work, we added preactivation to the model of Geyer and Herr^31^ through the addition of a new reflex based on HAM length. We then subjected the model to unexpected step-down perturbations and showed that preactivation markedly increases the robustness to unexpected step-down perturbations (see Fig. 1 for an overview of the used model, control strategy, and experimental setting). Furthermore, we scrutinized its biomechanical effects to derive a control strategy that can be used to naturally increase the tolerance of robotic systems towards step-down perturbations.

## Results

### Preactivation to different muscles

**Figure 2:**
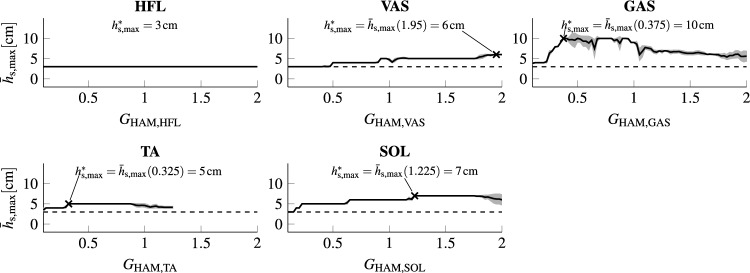
Maximally tolerated step height for preactivation of different muscles. Mean maximally rejected step height $\bar{h}_{\text {s,max}}$ (black) over 20 different initial body configurations per muscle for different levels of preactivation ($0.1 \le G_{\text {HAM,m}} \le 2.0$ in steps of 0.025). The mean absolute error $h_{\text {MAD}}$ for each gain value is depicted in gray. The dashed line indicates the performance of the original model ($h_\text {s} = {3}\,\textrm{cm}$). Results for HAM and GLU are not displayed, as any tested gain destabilized the model. $h^\text{*}_\text {s,max}$ is the maximally tolerated step height for preactivation of each muscle.

The effect of preactivation varied between the different muscles (see Fig. 2). A preactivation to HAM or GLU (not shown in Fig. 2) destabilized the model such that no stable gait on flat terrain was possible anymore. A preactivation to HFL did not destabilize the model but also did not improve the robustness of the model. Preactivation of GAS, SOL, TA, and VAS increased the robustness of the model. GAS preactivation resulted in the highest improvement with tolerated perturbations of up to $h_\text {s} = {10}\,\textrm{cm}$ and did not destabilize the model for any of the tested gains. That is, the model was able to reject step-down perturbations of up to $h_\text {s} = {3}\,\textrm{cm}$ for all tested gains. Preactivation of SOL allowed rejecting $h_\text {s} \le {7}\,\textrm{cm}$, of VAS $h_\text {s} \le {6}\,\textrm{cm}$, and of TA $h_\text {s} \le {5}\,\textrm{cm}$. Preactivation of SOL and VAS did not destabilize the model for any of the tested gains. For TA, higher gains destabilized the model even on flat terrain.

### Adaptations of gait

Preactivation to GAS improved the step rejection capabilities the most without decreasing the stability for any of the tested gains. Furthermore, GAS plays a prominent role in humans when coping with step-down perturbations^5,10,38^. Taking this together, we focused on the preactivation of GAS in order to analyze its effect on the resulting gait.

**Figure 3:**
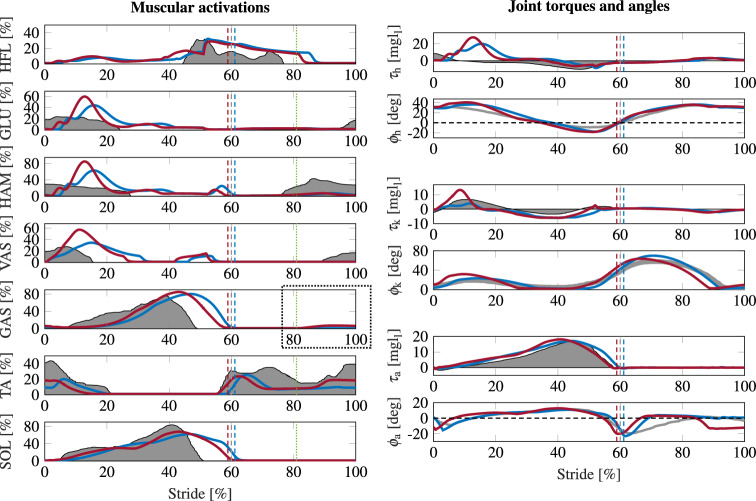
Walking on flat terrain. Muscular activations, joint torques, and angles of the model with preactivation of GAS (red), the original model of Geyer and Herr^31^ (blue), and experimental data (gray, adapted from Perry^17,31^) for hip, knee, and ankle. The data is depicted for one stride from heel-strike to heel-strike. Vertical dashed lines indicate toe-off, dotted lines (green) depict onset of preactivation. The dotted rectangle highlights preactivation of GAS.

Overall, the time course of the muscular activations did not markedly change by the added preactivation. The additional activation of GAS caused, through the original reflexes, an increase in activation of other muscles, namely of GLU, HAM, VAS, and TA (see Fig. 3). These are the four muscles that also show a preactivation during walking in humans^17^. We found activation of GLU, HAM, and VAS not in the preactivation phase where GAS activity increases, but rather after touch down in the early stance phase. TA activation was increased in the preactivation phase.

This combination of change in muscular activations led to a change in the ankle kinematics at the end of the swing phase (see $\phi _\text {a}$, Fig. 3). The foot was kept in a less dorsiflexed configuration, i.e., the toe was not lifted again at the end of swing but gradually approached the ground. At landing, the foot was almost horizontal (${4.2}^\circ$ vs. ${19.8}^\circ$) and the toes touched the ground shortly after the heel (8 ms, compared to 35 ms between heel and toe contact in the original model). This also influenced the ground reaction force, which was applied earlier with respect to heel strike.

The changed muscular activations increased the co-contraction at the different joints, which we measured by an increase in mean opposing torques $\text {CC}_{\tau _{\text {op}}, \text {j}}$. This was the case during early stance in the hip and knee joint and during late swing in the ankle joint. The mean opposing torques in the first 15% of the stride were increased by 45% (6.29 Nm vs. 4.35 Nm) at the hip and by 50% (39.98 Nm vs. 26.70 Nm) at the knee. The ankle joint experienced higher co-contraction in the late swing phase: during the last 15% of the stride cycle $\text {CC}_{\tau _{\text {op}}, \text {a}}$ was increased by 107% (2.28 Nm vs. 4.72 Nm). Also, the total torque at the hip ($\tau _\text {h}$) and the knee ($\tau _\text {k}$) in the early stance phase was increased, which led to greater displacements of these joints (see Fig. 3).

Additionally, the model took longer steps (average stride length ${1.72}\;\textrm{m}$ instead of ${1.59}\;\textrm{m}$), prolonging the swing phase (12% increase of swing phase duration and 1% increase of stance phase duration, see Table 1 for more details) and walked slightly faster (${1.41}\;{\tfrac{{\text{m}}}{{\text{s}}}}$ instead of ${1.37}\;{\tfrac{{\text{m}}}{{\text{s}}}}$). Furthermore, the amplitude of the center of mass (COM) vertical movement was increased (0.06 m instead of 0.04 m). The additional activation of several muscles increased muscle fatigue $\text {Mus}_{\text {Act}}$ by 7% (${0.49}\;{\frac{{\text{s}}}{{\text{m}}}}$ instead of ${0.46}\;{\frac{{\text{s}}}{{\text{m}}}}$) and cost of transport CoT by 16% (0.61 instead of 0.52).

**Table 1:** Average duration (*d*) and length (*l*) of entire stride (Str), duration of stance (St), swing (Sw) and double support (Ds) phase for the model without preactivation (Orig. Model) and the model with GAS preactivation (GAS PA).

	$d_\text {Str}$	$l_\text {Str}$	$d_\text {St}$	$d_\text {Sw}$	$d_\text {Ds}$
Orig. Model	1.16 s	1.59 m	0.71 s	0.45 s	0.13 s
Gas PA	1.23 s	1.72 m	0.72 s	0.50 s	0.11 s

### Reaction to step-down perturbations

**Figure 4:**
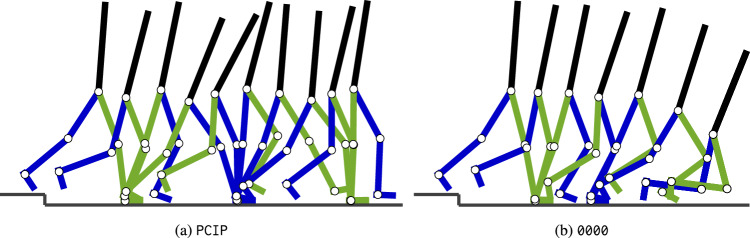
Step encounter ($h_{\text{s}} = {10}\,\textrm{cm}$) of the model with continuous preactivation (**a**) and the original model without preactivation (**b**). Snapshots are taken every 175 ms starting from the step encounter of the ipsilateral (green) leg. For (**b**), additionally, the final fall configuration is displayed. It can be seen that the contralateral foot (blue) comes to an early stop in (**b**) leading to the fall, whereas in (**a**), the model takes a long contralateral step and counters the fall. See also the supplementary material for videos of the complete step encounter of  (Video 1) and  (Video 2).

After the model with added preactivation reflex encountered the step-down perturbation, the contralateral foot performed a large step. With this, the model countered the forward momentum induced by the perturbation (see Fig. 4a). The original model without preactivation (see Fig. 4b) did not perform this large step and consequently fell.

**Figure 5:**
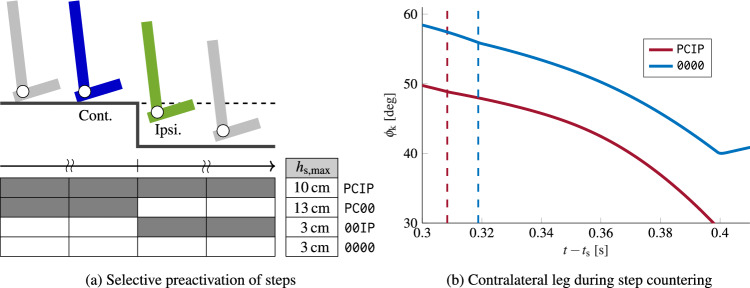
Determination of stabilization mechanism. (**a**) Robustness of continuous preactivation () and no preactivation () compared with conditions where preactivation of gastrocnemius was turned on (gray)/ off (white) selectively only until () or starting from () the step encounter. The highest step height $h_\text {s,max}$ until which rejection was continuously possible, was determined using increasing step heights. (**b**) Knee angle $\phi _\text{k}$ of the contralateral leg after the step encounter ($h_\text {s} = {10}\,\textrm{cm}$) of the ipsilateral leg at $t_\text {s}$. Dashed lines indicate the moment of early toe ground contact of the contralateral leg.  led to a fall, whereas with continuous preactivation (), the foot was lifted again and the step was rejected.

To analyze whether this large step caused the increased robustness and to understand the origin of the step-down perturbation response, we selectively turned on preactivation only until or starting from the step encounter. Namely, we determined the maximally rejected step height for a preactivation of all steps preceding the step encounter of the ipsilateral leg (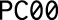) and for a preactivation added starting from the swing phase of the ipsilateral leg prior to the step encounter (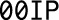, see also Fig. 5a). 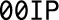 did not increase the robustness, whereas 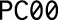 allowed countering perturbations of up to $h_\text{s} = {13}\,\textrm{cm}$, further highlighting the stabilizing role of the contralateral leg.

We, therefore, further analyzed the kinematics of the contralateral leg during the first step after the step encounter. Independent of preactivation (i.e., for 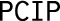 and 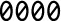), the model scuffed the contralateral foot during mid-swing, i.e., the toes shortly touched the ground. However, for the preactivated case (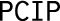), the ground reaction forces at this contact were smaller, and the foot was lifted again. This resulted in the large step compensating for the forward falling motion. Without preactivation (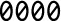), the leg directly transitioned into stance, leading to a very short step and therefore inducing the fall. This difference was caused by changed knee kinematics (see Fig. 5b). With preactivation (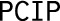), the knee joint angle $\phi _\text {k}$ of the contralateral leg during the scuffing was smaller, leading to a straighter leg around this contact. Therefore, the model could subsequently further straighten the knee to lift-off the foot again. Without preactivation (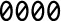), this was not possible without further colliding with the ground, leading to the observed early stance of the contralateral leg.

We further tested this stabilization mechanism by applying an additional torque to the contralateral knee during the last stride before the step encounter. With this, the original model (i.e., without preactivation) could reject a step of $h_\text {s} = {12}\,{\text{cm}}$, proving that the changed knee kinematics lead to a stabilization (see supplementary material, Video 3).

## Discussion

Reflexes are a key mechanism for humans to react to perturbations. NMS models can help to increase the understanding of the effect of specific reflexes and provide a biologically-inspired interface to robotic control. We studied the robustness of walking on rough and unknown terrain by subjecting the NMS walking model proposed by Geyer and Herr^31^ to unexpected step-down perturbations. The original model provides only limited robustness to unexpected step-down perturbations, and previously proposed improvements^36,37^ either require synchronization^37^ or early visual perturbation detection^36^. Here, we investigated a third option: improving robustness to step down perturbations based on additional proprioceptive feedback.

During locomotion, humans anticipate the moment of ground contact and prepare the swing leg for the impact using preactivation of muscles^6,10,17^. We show that preactivation can be integrated into an NMS model via an additional reflex, leveraging simple sensory information. In contrast to previously proposed adaptations^36,37,39^, we stay within the reflex-control scheme proposed by Geyer and Herr^31^. Our results show that the addition of preactivation of only one muscle increases the stability of the model considerably such that it can cope with markedly higher step-down perturbations ($h_\text {s} = {10}\,\textrm{cm}$ with GAS preactivation) than the original model of Geyer and Herr^31^ ($h_\text {s} = {3}\,\textrm{cm}$) and the model with the added feed-forward control of Haeufle et al.^37^ ($h_\text {s} = {7}\,\textrm{cm}$). Our adaptation does not drastically change the walking pattern. The muscular activations and joint torques mostly retain a time course similar to the original model.

The reflex can be implemented using length feedback of HAM. Alternatively, we also tested a more flexible implementation based on a fused sensor signal of foot height which can be tuned to allow rejection of even higher perturbations ($h_\text {s} = {12}\,\textrm{cm}$) when added to GAS (see supplementary material). Even though we cannot use these results to draw conclusions about the human neural control system, both of these feedback paths could be present in humans, representing different strategies related to adaptations due to age and disease (degeneration of sensor paths) but also to different environments. Either of these two different sensor signals can be used to implement preactivation in a robotic system.

The muscles for which we found an improvement all act either on the ankle or on the knee. GAS, which is acting on both of these joints, improved robustness most. The special role of GAS is confirmed by experimental results: GAS preactivation is reported in running and sometimes also in walking^40^, and in a study of the reaction to impact forces at heel strike^15^. Furthermore, GAS activation is adapted to react to step disturbances during running^10,38^ and walking^5^ and to different heights during drop jumping^41^. Here, the activation is timed to the expected time of ground contact^12^. Also, the foot configuration at touchdown tends towards a toe-first strategy, which is commonly used during stair walking and in unknown terrains^42,43^.

The increased robustness comes at the cost of additional muscular activations and therefore increased muscle fatigue and cost of transport. Not only is the preactivation of GAS added, but other muscles also show higher activations. Namely, preactivation of GAS leads to preactivation of TA, as also observed in humans^17^. HAM, VAS, and GLU show a higher activation at the beginning of the stance phase. All impacted muscles show a preactivation in humans during walking^17^.

Experimental results agree that preactivation causes co-contraction, which in turn leads to higher joint stiffness to prepare for the forthcoming impact^10–13^. In our study, we found some changes in the gait that adapt the landing and improve coping with the impact forces (higher co-contraction, more COM movement, less dorsiflexed landing, earlier GRF). However, the increased step rejection capabilities do not emerge from an improved landing at the step encounter. Instead, preactivation changes the knee kinematics to allow a reaction of the contralateral leg. The long step of the contralateral foot brings the COM more behind the base of support decreasing its velocity and preventing the fall. This step is enabled by preactivation, which influences the kinematics during the step encounter. Namely, the additional activation of muscles prevents an early ground contact of the contralateral foot by inducing a less bended knee. Besides changing the knee kinematics, preactivation also influenced other gait parameters, making it difficult to unveil the stabilizing mechanism. We therefore isolated the changed knee kinematics by applying a small additional torque $\tau _{\text{k}_\text{c},\text{add}}$ to the contralateral knee of the model without preactivation. $\tau _{\text{k}_\text{c},\text{add}}$ was only added during one stride and enabled the model without preactivation to reject a perturbation of $h_\text {s} = {12}\,\textrm{cm}$. This confirms that the stabilizing effect originates from the changed knee kinematics.

Our results emphasize preactivation as a simple and effective reflex-based accommodation strategy^4^ to increase robustness to step-down perturbations in an unknown environment. Furthermore, our results suggest that preactivation can also be used as an anticipatory strategy. Higher preactivation levels of GAS lead to toe-landing (commonly used by humans when stepping down^42,43^). Here, it is enough to preactivate the two strides prior to the step encounter to cope with step-down perturbations of up to $h_\text {s} = {10}\,\textrm{cm}$, pointing towards an anticipatory strategy. A detailed analysis of this strategy remains future work, as toe-landing is not in the scope of this study. If the perturbation is anticipated, the stabilization mechanism can also be implemented through an additionally applied knee torque $\tau _{\text{k}_\text{c},\text{add}}$.

We suggest that these insights can be used to improve bio-inspired controllers for bipedal robotic locomotion and can be incorporated into exoskeletons or orthosis. The proposed strategy does neither require a (visual) perturbation detection nor a synchronized internal model but can be implemented based on simple proprioceptive signals and is therefore well suited for a transfer to robotic systems. In future works, the stabilization mechanism should be ported to robotic systems to prove its generalizability, which was out of the scope of this study. Humans show preactivation of multiple muscles, however, to avoid the distortion of the stabilizing effect and obtain a simple strategy that can be ported to robotic systems, preactivation of multiple muscles was not considered in this study. Including further perturbations remains as future work, as, e.g. step-up perturbations can not be studied straightforwardly with the model due to its low ground clearance.

Overall, we showed that adding preactivation to an NMS model based on sensory information improves the model’s basin of attraction. We subsequently determined the underlying stabilization mechanism that increases the robustness to step-down perturbations. Namely, the markedly improved step rejection capabilities are due to changed knee kinematics. This opens up several possibilities to leverage the stabilizing effects of preactivation for bio-inspired controllers.

## Methods

### Walking model

We investigated the addition of preactivation to the NMS model proposed by Geyer and Herr^31^. This model predicts human walking in the sagittal plane and is composed of a trunk and two three-segmented legs (thigh, shank, foot), which are connected by six joints, each with a single rotational degree of freedom (hip, knee, and ankle for both legs, see Fig. 1). The joints are actuated by 7 Hill-type muscle-tendon units per leg: soleus (SOL), tibialis anterior (TA), gastrocnemius (GAS), vastus (VAS), hamstrings (HAM), gluteus (GLU) and hip flexors (HFL). Muscle stimulations are generated based on a number of reflexes that react to sensor information (trunk angle, ground contact, leg loading, joint angle, force and length information). Depending on the phase of the gait cycle (stance/ swing), different feedback paths are employed. The muscular activation predicted by this model is similar to human data.

### Preactivation

**Figure 6:**
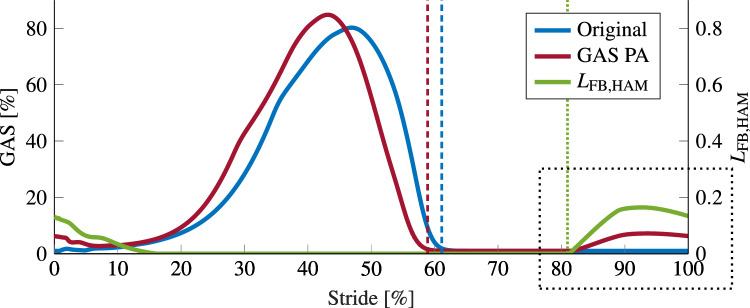
Feedback and gastrocnemius activation. The added preactivation reflex to gastrocnemius (GAS PA) using HAM length feedback $L_\text {FB,HAM}$ (green) induces an activation (red) at the end of the swing phase (dotted rectangle), which is not present in the original model (blue). Here, the gain of the reflex was set to $G_{\text {HAM,GAS}} = G^\text{*}_\text {GAS} = 0.375$. The data is displayed for one stride, from heel-strike to heel-strike. Dashed lines indicate toe-off (red/blue) and the dotted line depicts the onset of preactivation (green).

In our work, we added one additional reflex to the set of swing reflexes of the Geyer and Herr model: preactivation of muscles at the end of the swing phase. We implemented this preactivation on a proprioceptive basis by leveraging HAM length feedback. HAM length starts to increase in the last third of the swing phase and can therefore be used to prepare for ground contact of the swing leg. As proposed by Geyer and Herr^31^, it was calculated as:

$$\begin{aligned} L_{\text {FB,HAM}} = \frac{l_{\text {CE,HAM}}(t - \Delta t) - l_{\text {off,HAM}}}{l_{\text {opt, HAM}}}, \end{aligned}$$

where $l_{\text {CE,HAM}}$ is the HAM fiber length, $\Delta t = {5}\,\textrm{ms}$, $l_{\text {opt, HAM}} = {0.1}\;\textrm{m}$ and $l_{\text {off,HAM}}= {0.085}\,\textrm{m}$. In order to keep consistency with the original model, the values for $\Delta t$, $l_{\text {opt, HAM}}$, and $l_{\text {off,HAM}}$ were taken over from Geyer and Herr^31^. Changing the delay for the added reflex to up to 60 ms does not influence the main result. With a saturation to positive values, HAM length feedback is equal to 0 for most of the gait cycle, except for the time around ground contact (Fig. 6).

An additional length feedback path in the swing phase, from HAM to any muscle *m* of the same leg, thus generates a preactivation of the muscle *m*. To this end, the signal was gained with $G_{\text {HAM,m}}$ and added to the stimulation generated by the original model $S_{\text {m,orig}}$:

$$\begin{aligned} S_{\text {m}} = S_{\text {m,orig}} + G_{\text {HAM,m}} \cdot L_{\text {FB,HAM}}. \end{aligned}$$

This preactivation did not depend on knowledge of a possible perturbation but was present at the end of every swing phase. Figure 6 shows an exemplary activation of GAS with and without this additional reflex.

### Simulative evaluation

The model was implemented in Matlab Simulink R2021b. For all simulations, we used the ode15s solver (max. step size 10 ms) with a relative and absolute error tolerance of $10^{-5}$. The step-down perturbation was modelled as an unexpected disturbance, i.e., the model could not anticipate it. However, the horizontal position of the disturbance was chosen such that the model did not step on the edge of the step-down but always maintained flat ground contact (compare Haeufle et al.^37^ and Fig. 1).

#### Preactivation to different muscles

In order to evaluate the effect of a preactivation of each of the muscles, we added a reflex based on HAM length individually to each muscle *m* and assessed the maximal step height that the augmented model could reject. We tested the reflex over a range of gains ($0.1 \le G_{\text {HAM,m}} \le 2.0$, steps of 0.025) such that a maximal preactivation of around 40% of the maximal muscle activation could be obtained. This allowed covering the range of preactivation observed in humans (see Fig. 3). For each individual muscle *m* and gain $G_{\text {HAM,m}}$, we generated 20 randomized initial body configurations and tested whether the model could still walk on flat ground for 15 s. The configurations were obtained by introducing a random variation uniformly distributed between $\pm {0.05}\;\textrm{rad}$ ($\approx$ ${2.9^\circ }$) to each joint angle of the initial configuration used in Geyer and Herr^31^. We rejected $G_{\text {HAM,m}}$ if the model fell for any of the 20 configurations.

For the remaining gains, we then objected the model to increasing perturbation heights $h_\text {s}$, after $n_\text {s} = 10$ strides with a simulation length of 20 s. Starting from 0, $h_\text {s}$ was increased by increments of 1 cm until the model fell (i.e. the knee touched the ground) or joint limits were violated. For each value of $G_{\text {HAM,m}}$, we computed the mean $\bar{h}_{\text {s,max}}$ of the maximally rejected heights of the 20 initial configurations as well as the mean absolute deviation from the mean $h_{\text{MAD}}$ (see Fig. 2). For each muscle *m*, we then determined the robustly achievable maximal step height $h_{\text {s,max}}^\text{*} = \text{max}(\bar{h}_{\text {s,max}})$ and recorded the corresponding optimal gain $G^\text{*}_\text{m}$. If several gains achieved the same performance, we recorded the smallest gain.

The results showed some sensitivity to the initial configurations for $G_{\text {HAM,TA}}$, $G_{\text {HAM,SOL}}$, and $G_{\text {HAM,GAS}}$. For all three muscles, this sensitivity occurred for higher gains than the identified optimal gain and did thus not influence the results. A closer analysis of the first sensitive region for $G_{\text {HAM,GAS}}$, furthermore revealed that between 0.4 to 0.7, the model changed from landing with the heel to toe-landing. Thus, the sensitivity results from the limited foot model: as the foot model is restricted to two contact points, small differences in solver steps lead to markedly different results (i.e., heel- vs. toe-landing), explaining the observed sensitivity.

#### Adaptations of gait

For the best achievable disturbance rejection with $G_{\text {HAM,GAS}} = G^\text{*}_{\text {GAS}} = 0.375$, we compared the model with preactivation to the original model of Geyer and Herr^31^ and to experimental data from Perry^17^. We compared the muscular activations as well as the joint torques and angles during one stride. To evaluate the overall change in muscular activation, we calculated muscle fatigue^44^ for 50 strides:

$$\begin{aligned} \text{Mus}_{\text{Act}} = \frac{1}{\text{distance}}\int _{t_{\text{start}}}^{t_{\text{end}}} [\sum _{\text{m}} A_{\text{m}}^\text{2}(t)]dt \end{aligned}$$

with $t_{\text{start}}, t_{\text{end}}$ being the start and end time of the 50 strides’ time interval and $A_\text{m}(t)$ the activation of muscle *m*. We also calculated the cost of transport for 50 strides based on positive and negative mechanical work^45,46^:

$$\begin{aligned} W^\text{+}&= \int _{t_{\text{start}}}^{t_{\text{end}}} \left[ \sum _{\text{j}} \text{max}(\tau _{\text{j}}(t) \cdot \omega _{\text{j}}(t),0) \right] dt\\ W^\text{-}&= \int _{t_{\text{start}}}^{t_{\text{end}}} \left[ \sum _{\text{j}} \text{min}(\tau _{\text{j}}(t) \cdot \omega _{\text{j}}(t),0) \right] dt\\ \text{CoT}&= \frac{1}{\text{distance}\cdot \text{bodyweight}} \left( \frac{W^\text{+}}{0.25} + \frac{W^\text{-}}{-1.2} \right) \end{aligned}$$

with $\tau _{\text{j}}(t), \omega _\text{j}(t)$ being torque and angular velocity of joint *j*. To further characterize the gait, we calculated speed, stride length and duration, as well as stance, swing, and double support duration from the average of 50 strides. We additionally analyzed co-contraction by comparing the torques applied at each joint. Namely, if the co-contraction of two muscles is increased, the torques applied in both directions of the joint increase, while the net torque stays the same. To quantify co-contraction, we, therefore, compared the mean opposing torques that equalize each other at a joint *j* during the first and last 15% of the gait cycle:

$$\begin{aligned} \text{CC}_{\tau _{\text{op}}, \text{j}} = \frac{1}{t_\text{1}-t_\text{0}}\int _{t_{\text{0}}}^{t_{\text{1}}} \text{min} \left( \left| \tau ^\text{+}_{\text{j}}(t) \right| , \left| \tau ^\text{-}_{\text{j}}(t) \right| \right) dt \end{aligned}$$

with $[t_{\text{0}}, t_{\text{1}}] = \{[0,15],[85,100]\}$% of the gait cycle and $\tau ^\text{+}_{\text{j}}(t), \tau ^\text{-}_{\text{j}}(t)$ the torques applied in positive and negative direction of the joint.

#### Reactions to step-down perturbations

To further identify the mechanism that helps to reject the step perturbation, we analyzed the kinematics during the step rejection and selectively turned on preactivation before/after the step. As in the previous section, we consider a preactivation to GAS with $G_{\text {HAM,GAS}} = G^\text{*}_{\text {GAS}} = 0.375$. For the analysis of the kinematics, we took snapshots every 175 ms starting from the step encounter of the ipsilateral leg ($h_\text {s} = {10}\,\textrm{cm}$) for the model with preactivation and the original model. We also compared the trajectory of the knee angle of the contralateral leg after the step encounter, around the moment of foot scuffing. The relevant change in knee kinematics was tested by adding an additional torque. Namely, we used the original model without preactivation and applied an additional torque $\tau _{\text{k}_\text{c},\text{add}} = {-0.6}\;{\textrm{mgl}_\text{l}}$ to the contralateral knee during the last stride before the step encounter.

To evaluate whether the stabilizing effect of preactivation results from the adapted landing of the ipsilateral leg or from an adaptation of the contralateral leg, we compared the step rejection capabilities of the following conditions:

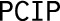
—continuous preactivation

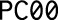
—preactivation until landing

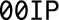
—preactivation from landing

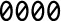
—no preactivation

(nomenclature: 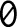: not preactivated, 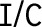: ipsi-/contralateral leg preactivated, 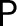: all steps before/after preactivated). We followed the same protocol as described above to determine the maximally rejected step height. The resulting $h_\text {s,max}$ did not differ for the 20 different initial configurations, except for 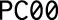, where, in some cases, even higher steps (up to $h_\text {s,max} = {15}\,\textrm{cm}$) could be rejected. We report the maximal step size that was rejected in all cases.

### Supplementary Information

Supplementary Information 1.

Supplementary Video 1.

Supplementary Video 2.

Supplementary Video 3.

Supplementary Video 4.

## Data Availability

The experimental results are available at 10.18419/darus-3492. The model will be shared upon request to the authors with the consent of the author of the original unmodified model^31^.
