# Supplementary material for: Bioinspired preactivation reflex increases robustness of walking on rough terrain
Source: Sci Rep. 2023 Aug 14;13:13219. doi: 10.1038/s41598-023-39364-3 (PMC10425464; doi:10.1038/s41598-023-39364-3)
Supplement: Supplementary file 1 — Supplementary Information 1. [file 41598_2023_39364_MOESM1_ESM.pdf]

Supplementary Material: Bioinspired Preactivation Reflex Increases Robustness of Walking on Rough Terrain

Elsa K. Bunz[1,2,*], Daniel F. B. Haeufle[3,4,5], C. David Remy[2,5,6], and Syn Schmitt[1,2,5]

[1]Institute for Modelling and Simulation of Biomechanical Systems, University of Stuttgart, Stuttgart, Germany
[2]Stuttgart Center for Simulation Science, University of Stuttgart, Stuttgart, Germany
[3]Institute of Computer Engineering, Heidelberg University, Heidelberg, Germany
[4]Hertie Institute for Clinical Brain Research and Center for Integrative Neuroscience, Tuebingen, Germany
[5]Center for Bionic Intelligence Tuebingen Stuttgart, Tuebingen Stuttgart, Germany
[6]Institute for Nonlinear Mechanics, University of Stuttgart, Stuttgart, Germany
[*]elsa.bunz@imsb.uni-stuttgart.de

Sensory origin of preactivation - Difference of foot heights

Preactivation of muscles can not only be achieved by using a basal length feedback but also by a more fused and flexible signal. Namely, the time before ground contact can be estimated using sensory information about limb positions. If the terrain is expected to be flat, the difference of foot heights between the swing and the stance foot provides information about the height of the swing foot above the ground. Using the height of the swing foot as sensory feedback requires the fusion of sensor signals and an internal model[1], however, it offers a lot of possibilities to adapt the preactivation (i.e. the duration and the course of time) e.g. to different terrains. Also, for a (torque-actuated) robotic system, foot height is a more natural sensor signal than muscle length. Therefore, we use foot as an alternative feedback signal to evoke preactivation and test whether it also improves the robustness to unexpected step-down perturbations.

The stimulation is calculated in three steps:

$$h_{\mathrm{Sw}} = y_{\mathrm{Sw}} - y_{\mathrm{St}}$$

$$H_{\mathrm{FB,m}} = \frac{S_{0,\mathrm{m}}}{\sigma_{\mathrm{m}}\sqrt{2\pi}}\, e^{\frac{-(h_{\mathrm{Sw}}-\mu_{\mathrm{m}})^2}{2\sigma_{\mathrm{m}}^2}}$$

$$S_{\mathrm{m}} = S_{\mathrm{m,orig}} + G_{\mathrm{H,m}} H_{\mathrm{FB,m}}$$

where y denotes the y-position of the center of gravity of the swing (Sw) and stance foot (St) and m specifies the considered muscle. $S_{0,\mathrm{m}}$ is the prestimulation as in Geyer and Herr[2] and $S_{\mathrm{m,orig}}$ is the stimulation of the muscle using the reflexes of the original model. The three parameters $G_{\mathrm{H,m}}$, μ_{m}, σ_{m} allow influencing the shape of the additional stimulation and therefore provide a greater range of possible stimulations than HAM length feedback.

We performed a manual tuning of the three parameters $G_{\mathrm{H,GAS}}$, μ_{GAS}, σ_{GAS} in order to obtain good step rejection capabilities. The final tuning was $G_{\mathrm{H,GAS}} = 1.3\,\mathrm{m}$, $\mu_{\mathrm{GAS}} = 0.005\,\mathrm{m}$, $\sigma_{\mathrm{GAS}} = 0.02\,\mathrm{m}$. This changes the activation of gastrocnemius at the end of the swing phase, as can be seen in Supplementary Figure S1. As with the HAM length feedback, this additional reflex stabilizes the model in the face of step-down perturbations and the highest tolerated step-down perturbation is further increased, namely to $h_{\mathrm{s}} \leq 12\,\mathrm{cm}$. This is an improvement of 400 % to the original model, while keeping very similar gait characteristics. Video 4 shows the kinematics of the preactivated model and its reaction to a step of $h_{\mathrm{s}} = 12\,\mathrm{cm}$.

Videos

We provide four videos showing a visualization of the model kinematics to illustrate the behavior of the model around the step encounter:

- **`Video 1`**
  This video shows the model with added preactivation to GAS via length feedback from HAM with $G_{\mathrm{HAM,GAS}} = G^{*}_{\mathrm{GAS}} = 0.375$ (condition `PCIP`). The model is confronted with and rejects a step of $h_{\mathrm{s}} = 10\,\mathrm{cm}$.

- **`Video 2`**
  This video shows the original model of Geyer and Herr[2] (condition `0000`) encountering a step of 10 cm, after which it falls.

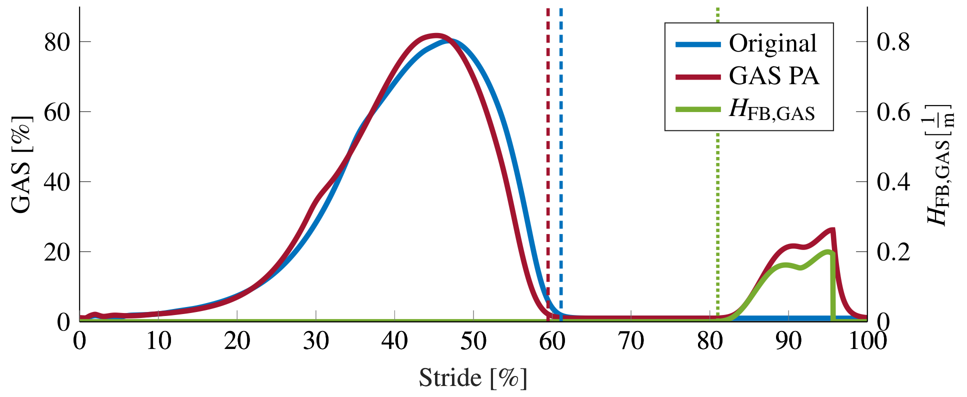

Supplementary Figure S1. Feedback and gastrocnemius activation. The added preactivation reflex to gastrocnemius (GAS PA) using foot height feedback $H_{FB,GAS}$ (green, $G_{H,GAS} = 1.3\,m$) induces a preactivation of GAS at the end of the swing phase (red), which is not present in the original model (blue). The data is displayed for one stride, from heel-strike to heel-strike, dashed lines indicate toe-off (red/blue), the dotted line depicts the onset of preactivation (green).

- **`Video 3`**
  This video shows the model without any added preactivation, but an additional torque $\tau_{k_c,add} = -0.6\,mgl_l$ applied at the contralateral knee (blue) during the last stride before the step encounter. With this addition, the model can reject a step of $h_s = 12\,cm$.

- **`Video 4`**
  This videos shows the model with an added preactivation to GAS by height feedback ($G_{H,GAS} = 1.3\,m$, $\mu_{GAS} = 0.005\,m$, $\sigma_{GAS} = 0.02\,m$) coping with a step of $h_s = 12\,cm$.

In all videos the camera view point is following the hip. The total size of the four videos is 1.2 MB. The videos can be played using standard multimedia players like VLC media player (tested with V3.0.9.2).

Contact

In case of questions, please contact Elsa K. Bunz (elsa.bunz@imsb.uni-stuttgart.de).

References

1. Kurtzer, I. L., Pruszynski, J. A. & Scott, S. H. Long-latency reflexes of the human arm reflect an internal model of limb dynamics. *Curr. Biol.* **18**, 449–453, DOI: 10.1016/j.cub.2008.02.053 (2008).

2. Geyer, H. & Herr, H. A muscle-reflex model that encodes principles of legged mechanics produces human walking dynamics and muscle activities. *IEEE Transactions on Neural Syst. Rehabil. Eng.* **18**, 263–273, DOI: 10.1109/TNSRE.2010.2047592 (2010).
